# Hexavalent Chromate Reductase Activity in Cell Free Extracts of *Penicillium* sp.

**DOI:** 10.1155/2013/909412

**Published:** 2013-08-21

**Authors:** Damaris L. Arévalo-Rangel, Juan F. Cárdenas-González, Víctor M. Martínez-Juárez, Ismael Acosta-Rodríguez

**Affiliations:** ^1^Universidad Autónoma de San Luis Potosí, Facultad de Ciencias Químicas, Centro de Investigación y de Estudios de Posgrado, Laboratorio de Micología Experimental Avendia Dr. Manuel Nava No. 6, Zona Universitaria, 78320 San Luis Potosí, SLP, Mexico; ^2^Área Académica de Medicina Veterinaria y Zootecnia, Instituto de Ciencias Agropecuarias, Universidad Autónoma del Estado de Hidalgo, Zona Universitaria, Rancho Universitario Km 1, 43600 Tulancingo de Bravo, HGO, Mexico

## Abstract

A chromium-resistant fungus isolated from contaminated air with industrial vapors can be used for reducing toxic Cr(VI) to Cr(III). This study analyzes in vitro reduction of hexavalent chromium using cell free extract(s) of the fungus that was characterized based on optimal temperature, pH, use of electron donors, metal ions and initial Cr(VI) concentration in the reaction mixture. This showed the highest activity at 37°C and pH 7.0; there is an increase in Cr(VI) reductase activity with addition of NADH as an electron donor, and it was highly inhibited by Hg^2+^, Ca^2+^ and Mg^2+^, and azide, EDTA, and KCN.

## 1. Introduction

Chromium (Cr) toxicity is one of the major causes of environmental pollution emanating from tannery effluents. This metal is used in the tanning of hides and leather, the manufacture of stainless steel, electroplating, and textile dyeing and used as a biocide in the cooling waters of nuclear power plants, resulting in chromium discharges causing environmental concerns [1]. Cr exists in nine valence states ranging from −2 to +6. Of these states, only hexavalent chromium [Cr(VI)] and trivalent chromium [Cr(III)] have primary environmental significance because they are the most stable oxidation forms in the environment [2]. Both are found in various bodies of water and wastewaters [3]. Cr(VI) typically exists in one of these two forms: chromate (CrO_4_^−2^) or dichromate (Cr_2_O_7_^−2^), depending on the pH of the solution [3]. These two divalent oxyanions are very water soluble and poorly adsorbed by soil and organic matter, making them mobile in soil and groundwater [2]. Both chromate anions represent acute and chronic risks to animals and human health since they are extremely toxic, mutagenic, carcinogenic, and teratogenic [4]. In contrast to Cr(VI) forms, the Cr(III) species, predominantly hydroxides, oxides, or sulphates, are less water soluble, mobile (100 times less toxic) [5], and (1,000 times less) mutagenic [6]. The principal techniques for recovering or removing Cr(VI), from wastewater are chemical reduction and precipitation, adsorption on activated carbon, ion exchange, and reverse osmosis, in a basic medium [7]. However, these methods have certain drawbacks, namely, high cost, low efficiency, and generation of toxic sludge or other wastes that require disposal and imply operational complexity [8].

An alternative to these methods is the removal of heavy metal contaminants by microorganisms. The metal removal ability of microorganisms, including bacteria [2, 6, 8, 9], microalgae [7, 10], and fungi [1, 11], has been studied extensively. Fungi, in general, are well known for their ability to biosorb and bioaccumulate metals [1, 11, 12] and have also been reported to be involved in reduction (biotransformation) of Cr(VI) to Cr(III) form [11–13]. The common Cr(VI) detoxification mechanisms reported in Cr-resistant microorganisms are periplasmic biosorption and intracellular bioaccumulation and biotransformation through direct enzymatic reaction [14, 15] or indirectly with metabolites [16]. In Cr(VI)-resistant filamentous fungi, such as *Paecilomyces *[13], *Aspergillus* and *Penicillium* [17], and *Trichoderma* [18], the Cr(VI) detoxification through transformation of Cr(VI) to Cr(III) form was observed due to cellular metabolism processes based on the reducing power of carbon sources. On the other hand, bioreduction of Cr(VI) has been demonstrated in several bacterial species including *Pseudomonas* sp. [19], *Escherichia coli* [20], *Bacillus* sp. [21], *Desulfovibrio* sp. [22], *Microbacterium* sp. [23], and *Shewanella* sp. [24], some fungi like *A. niger *and *A. parasiticus* [11], *Paecilomyces *[13], *Fusarium* sp. [25], *Paecilomyces lilacinus* [26], and *Hypocrea tawa* [27], and the yeasts *Candida maltosa* [28], *Pichia* sp. [29] and *Candida utilis* [30]. Direct microbial reduction of Cr(VI) to Cr(III) is the most promising practice with proved expediency in bioremediation. 

The objective of this study was to analyze in vitro reduction of Cr(VI) by cell free extracts of *Penicillium *sp. resistant to Cr(VI). 

## 2. Experimental

### 2.1. Microorganism and Culture Conditions

A chromate-resistant filamentous fungus was isolated from polluted air with industrial vapors, near the Chemical Science Faculty, located in the city of San Luis Potosí, Mexico, in Petri dishes containing modified Lee's minimal medium (LMM, [31]) (with 0.25% KH_2_PO_4_, 0.20% MgSO_4_, 0.50% (NH_4_)_2_SO_4_, 0.50% NaCl, and 0.25% glucose) supplemented with 500 mg/L K_2_CrO_4_; the pH of the medium was adjusted and maintained at 5.3 with 100 mMol/L citrate-phosphate buffer. The cultures were incubated at 28°C for 7 days. The strain was identified based on its morphological structures such as the color, diameter of the mycelia, and microscopic observation of formation of spores [32]. Fungal cultures grown in thioglycolate broth were used as primary inoculums.

### 2.2. Cr(VI) Reduction by Resting Cells of *Penicillium* sp

Culture suspensions of *Penicillium *sp. were grown for 4 days in 100 mL thioglycolate broth (pH 7.0) and harvested by centrifugation at 3000 ×g at 4°C; cell pellets (10 mL) obtained on centrifugation were washed twice with 100 mM potassium phosphate buffer (pH 7.0) and resuspended in the same buffer. Triplicates of these suspended cell pellets were spiked with Cr(VI) concentrations of 2–10 mg/100 mL, vortexed for 30 min, and incubated at 30°C for 6 h. Heat-killed (2 mL) culture pellets were used as control. After 6 h incubation, the tubes were centrifuged, and 100 *µ*L aliquots were withdrawn from each sample to estimate the remaining Cr(VI) by 1,5-diphenyl carbazide (DPC) method [33].

### 2.3. Cr(VI) Reduction by Permeabilized Cells of *Penicillium* sp

Bacterial culture of *Penicillium* sp. was grown for 4 days, harvested, and washed with potassium phosphate buffer (pH 7.0) as described above. The suspended culture pellets were treated with 0.2% (w/v) sodium dodecyl sulphate, 0.2% tween 80, (v/v), 0.2% Triton X-100 (v/v), and 0.2% toluene (v/v), by vortexing for 30 min to achieve cell permeabilization. Permeabilized cell suspensions (0.5 mL) were then added with 2–10 mg/100 mL of Cr(VI) as final concentrations and incubated for 6 h at 30°C. Experiments with each set of permeabilization treatment and Cr(VI) concentrations were performed in triplicates.

### 2.4. Preparations of Cell-Free Extracts

Cell-free extracts (CFE) of *Penicillium* sp. were prepared by modifying the previously published protocols [34]. Fungal suspensions grown for 4 days in 400 mL thioglycolate broth were harvested at 3000 ×g at 4°C for 10 min, washed, and resuspended in 100 mM potassium phosphate buffer (pH 7.0). The culture pellets thus obtained were resuspended in the 5% (v/v) of the original culture volume in 100 mM potassium phosphate buffer (pH 7.0). These cell suspensions were placed in ice bath and disrupted using an Ultrasonic Mini Bead Beater Probe (Densply) with 15 cycles of 60 sec for each one. The sonicate thus obtained was then centrifuged at 3000 ×g for 10 min at 4°C. The pellet was resuspended in 100 mM potassium phosphate buffer (pH 7.0, and this is the CFE).

### 2.5. Chromate Reductase Assay

Enzymatic chromate reduction was estimated as described previously using a standard curve of Cr(VI) 0–30 mM [34]. Assay mixtures were modified from those described in previous studies [34]. The reaction system (1.0 mL) was made up of varying Cr(VI) final concentrations (5–30 mM) in 700 *µ*L of 100 mM potassium phosphate buffer (pH 7.0) added with 250 *µ*L aliquots of CFE for chromate reduction and 50 *µ*L of NADH. The system volume of 1.0 mL was kept constant for all experiments. 

Chromate reductase activity was measured at 37°C at different pH values using several buffers (100 mM phosphate-citrate, pH 5.0; 50 mM phosphate, pH 6.0–8.0, and 50 mM TRIS-HCl, pH 8-9). The effect of temperature was studied by measuring chromate reductase activity at different incubation temperatures between 20 and 60°C, at pH optimum. The CFE samples were also treated with several metal ions to a final concentration of 1 mM at optimal pH and temperature; Na^+^, Ca^2+^, Cu^2+^, Hg^2+^, Mg^2+^, Cd^2+^, and Fe^3+^ were tested by using 10 mM solutions of Na_2_SO_4_, CaCl_2_, CuCl_2_, HgCl_2_, MgCl_2_, CdCl_2_, and FeCl_3_. The electron donors tested were NADH, glucose, sodium acetate, formic acid, citrate, cystin, lactic acid, and ascorbic acid in a final concentration of 1 mM, and the inhibitors were EDTA, KCN, NaN_3_, and *β*-mercaptoethanol at the same concentration. Unit enzyme activity for chromate reductase was derived as amount of enzyme that reduces 1 mMol of Cr(VI) per min at 37°C. Specific activity was defined as unit chromate reductase activity per minute per mg protein concentration in the CFE. Protein concentrations were estimated using Lowry method [35]. 

## 3. Results and Discussion

### 3.1. Cr(VI) Removal by Resting Cell of *Penicillium* sp

**Figure 1:**
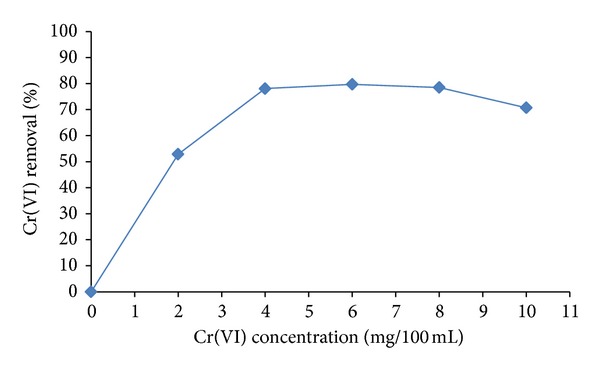
Resting cell assays for Cr(VI) reduction by *Penicillium* sp. performed at initial concentrations of 0–10 mg/100 mL of Cr(VI), pH 7.0 and 37°C.

The resting cells of the fungus were expedient in reducing 0–10 mg/100 mL Cr(VI) concentrations in 8 hours as shown in Figure 1. The fungus removal was between 53% and 70% (2–10 mg/100/mL) of the metal, and these results resemble those reported by *A. niger* and *A. parasiticus* [11] *Fusarium solani *[25], *Paecilomyces lilacinus* [26], and the bacteria *Pseudomonas *sp. [19]. Structural properties of the biosorbent including the cellular support and other several factors are known to affect the biosorption rate [36].

### 3.2. Cr(VI) Reduction by Permeabilized Cells of *Penicillium* sp

**Figure 2:**
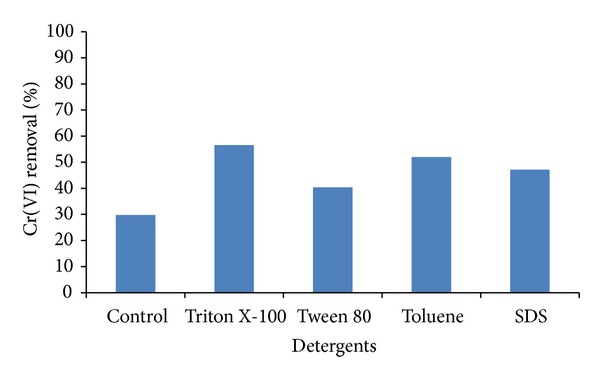
Permeabilized cell assays for Cr(VI) reduction by *Penicillium* sp. performed at initial concentrations of 28 mM of Cr(VI), pH 7.0 and 37°C.

The cell permeabilization increased the Cr(VI) reduction by the resting cells, as the permeabilized cells with Triton X-100 which could reduce 57%, Toluene 52%, SDS 47.4%, and Tween 80, 40.4% (Figure 2) of 30 mM Cr(VI) within 6 h, suggesting an efficient intracellular mechanism of chromate reduction. The Cr(VI) reductase activity in CFE of cells grown in absence of Cr(VI) was 94.07 *µ*moles/min/mg protein. These results indicate that the Cr(VI) reductase was associated with the CFE. Fungal, yeast, and bacteria chromate reductases have been localized either in CFE of *A. niger* and *A. parasiticus* [11], *Pichia jadinii* M9, and *Pichia anomala *M10 [37],* Pichia *sp. [29],* and Bacillus* sp. [21], and cytosolic fraction of *C. maltosa* [28], *Pichia* sp. [29], and *Pannonibacter phragmitetus* [38] and membrane fraction *Pseudomonas* sp. G1DM21 [19],* Bacillus megaterium* [39], and *Enterobacter cloacae* [40].

### 3.3. Effect of pH on the Chromate Reductase Activity

**Figure 3:**
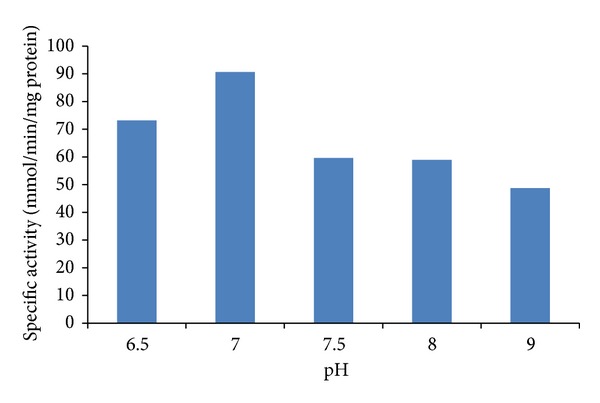
Effect of pH on Cr(VI) reductase activity in cell-free extracts of *Penicillium *sp. determined in different buffers (pH 6.5–9.0) with initial concentration of 5.6 mM Cr(VI), at 37°C.

The functioning of the chromate reductase of *Penicillium* sp. was characterized in different *in vitro* conditions. To define the optimal pH, the Cr(VI) reductase assays were carried in potassium phosphate, citrate phosphate, and Tris-HCl buffers of differential pH ranges; of the different buffers used the potassium phosphate buffer showed a characteristic pH curve for the enzymatic activity with an optimum pH of 7.0, as depicted from Figure 3, and these results resemble those reported by the fungal *A. niger* and *A. parasiticus* [11] and the yeast *P. jadini* M9 [37]. Other authors reported stability between 7.0 and 7.4 for the bacteria *Pseudomonas* sp. G1DM21 [19], 6.5 and 7.5 in *E. coli* CFE [41], and in the range of 5.0 to 8.0 in *Bacillus* sp. [42].

### 3.4. Effect of Temperature on the Chromate Reductase Activity

**Figure 4:**
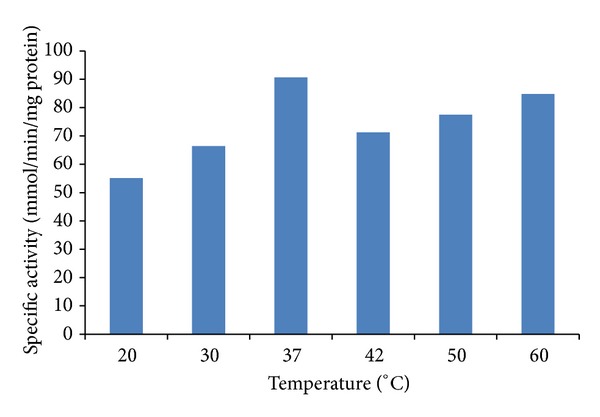
Effect of temperature on Cr(VI) reductase activity in cell-free extracts of *Penicillium* sp. with initial concentrations of 28 mM Cr(VI) at pH 7.0.

The optimal temperature for the Cr(VI) reductase activity was 37°C, but the reductase activity was altered significantly at 20°C (39% of inhibition), but when the assays were performed at 50°C temperature the reductase activity we found 14.2% of inhibition Figure 4. For *P. jadinii* M9, incubation at 55°C produced a reduction in activity of 55% [37]. In *P. anomala* when incubated at 8°C, a decrease in activity of 25% was observed, and at 50°C the activity was 50%. For *A. niger* and *A. parasiticus* [11], *Pseudomonas *sp. G1DM21 [19], *E. coli *a [41], and *Bacillus* sp. CFEs [42], the thermal stability was of 30°C [41, 42]. On the contrary, *Pseudomonas putida* CFE probed to be more resistant, keeping its stability up to 50°C [43].

### 3.5. Effect of Different Metal Cations on the Chromate Reductase Activity

**Figure 5:**
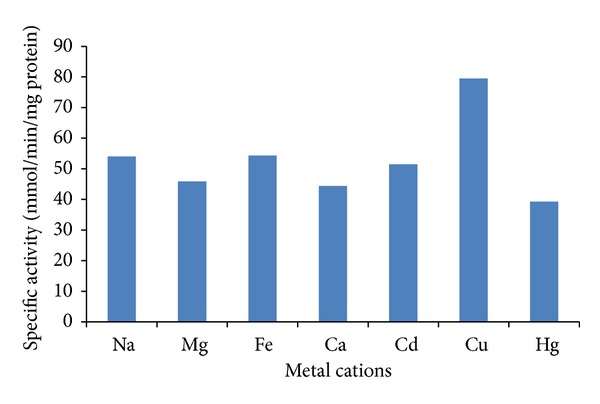
Effect of different metal cations on Cr(VI) reductase activity in cell-free extracts of *Penicillium* sp. at pH 7.0 and 37°C.

The effect of different metal cations on the chromate reductase activity of *Penicillium* sp. was determined as exhibited in Figure 5. All the metal ions tested inhibit the Cr(VI) reductase activity of the CFE of 12% with Cu^2+^ and 40.2% with Na^+^, and these results are different than those reported by the yeast *P. jadinii* M9 Chromate reductase because only Cu^2+^ and Na^+^ produced an augmentation in the activity of 63 and 30%, respectively [37], and all other ions tested had an inhibitory effect but in different levels. A decrease of 56.5% was observed with Hg^2+^, while addition of Mg^2+^, Fe^3+^, Ca^2+^, and Cd^2+^ resulted in a decrease of activity between 40% and 51%. In *P. anomala* M10 chromate reductase, only Cu^2+^ produced a raise in activity of a 31%. Inhibition by Hg^2+^ was higher in *P. anomala *and* Pseudomonas *sp. than in* Penicillium* sp. with a decrease in activity of 85% and 90%, respectively [19, 37]. Inhibition by Ca^2+^ and Mg^2+^ was approximately 50%, while Fe^3+^ reduced the activity by 32%. These results agree with those reported for *Arthrobacter crystallopoietes* [44], and *Bacillus* sp. [42]. On the other hand, inhibition by Hg^2+^ can be related to its affinity to –SH ligands, then suspecting the presence of this chemical group in the active site of the enzyme related to chromate reductase activity [44].

### 3.6. Effect of Different Electron Donors on the Chromate Reductase Activity

**Figure 6:**
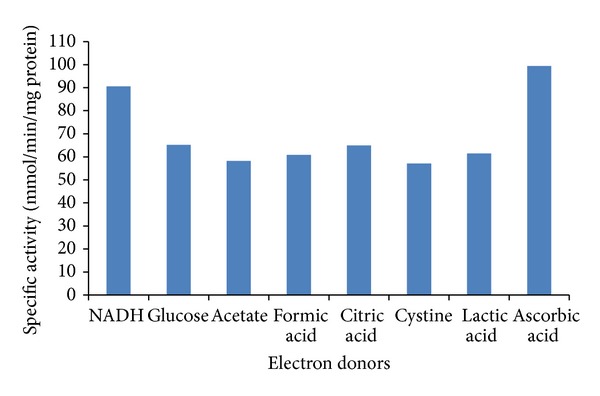
Effect of different electron donors on Cr(VI) reductase activity in cell-free extracts of *Penicillium* sp. at pH 7.0 and 37°C.

The reductase activity increased on supplementation in the reaction mixtures with electron donors. All the electron donors analyzed increased the activity, and the most efficient were ascorbic acid, NADH, glucose, and citrate by 4.4, 4.0, 2.9, and 2.87 times, respectively (Figure 6), and these results are like those reported by the yeasts *P. jadinii* M9 and *P. anomala* Chromate reductase with NADH [37] and *Pseudomonas* sp. with citrate, acetate, glucose, and formate [19]. In previous reports on *Bacillus* sp., glucose has been reported to act as an electron donor and has been demonstrated to increase Cr(VI) reduction [45, 46], and also formate-dependent Cr(VI) reductases have been reported in *Shewanella putrefaciens* MR-1 [47]. Our work supports other studies reporting NADH or NADPH-dependent enzymatic reduction of Cr(VI) under aerobic conditions [19, 20, 37, 42, 43]. According to Ramirez-Díaz et al. [48], the oxidation of NADH donates an electron to the chromate reductase enzyme, and then the electron is transferred to Cr(VI) reducing it to the intermediate form, Cr(V), which finally accepts two electrons from other organic substances to produce Cr(III).

### 3.7. Effect of Different Respiratory Inhibitors on the Chromate Reductase Activity

**Figure 7:**
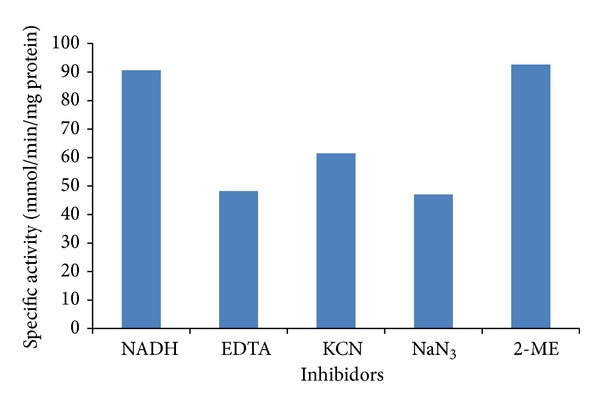
Effect of different inhibitors on Cr(VI) reductase activity in cell-free extracts of *Penicillium* sp. at pH 7.0 and 37°C.

 Respiratory inhibitors like azide (1 mM), EDTA (1 mM), and cyanide (1 mM) caused inhibitions of 48%, 47%, and 32%, respectively (Figure 7), in the Cr(VI) reductase activity; these results corroborate with those obtained in previous studies, and it has been observed that cyanide and azide partially inhibited purified chromate reductase of *E. coli* ATCC 33456 19, [20] and aerobic chromate reduction by *Bacillus subtilis* [49] and inhibited more than 50% of membrane associated chromate reductase activity of *S. putrefaciens* MR-1 [49] while no inhibition was observed in CFE of *Bacillus *sp. ES29 [44]. Respiratory inhibitors act on de novo protein synthesis or affect the respiratory chain intermediates responsible for Cr(VI) reduction, wherein Cr(VI) serves as a terminal electron acceptor [43]. 

## 4. Conclusion

The present study analyzed a very efficient Cr(VI) reductase of *Penicillium* sp. Chromate reductase assays of the cell-free extracts (CFE) have shown a high Cr(VI) reductase activity. The Cr(VI) reduction potential of the resting cells was increased by cell permeabilization. Optimum temperature and pH of chromate reductase activity of the bacterium were found to be 37°C and 7.0, respectively, and activity was enhanced in presence of 0.1 mM NADH and other electron donors. 1 mMol of metal ions like Cu^2+^, Na^+^, Hg^2+^, Mg^2+^, Fe^3+^, Ca^2+^, and Cd^2+^ and respiratory inhibitors resulted in a decrease of the activity. Finally, the results of higher rates of Cr(VI) reduction by the CFE, functionality of the Cr(VI), indicate a potential application of *Penicillium* sp. for Cr(VI) bioremediation.
